# LpMab-12 Established by CasMab Technology Specifically Detects Sialylated *O*-Glycan on Thr52 of Platelet Aggregation-Stimulating Domain of Human Podoplanin

**DOI:** 10.1371/journal.pone.0152912

**Published:** 2016-03-31

**Authors:** Yukinari Kato, Satoshi Ogasawara, Hiroharu Oki, Polina Goichberg, Ryusuke Honma, Yuki Fujii, Mika K. Kaneko

**Affiliations:** 1 Department of Regional Innovation, Tohoku University Graduate School of Medicine, 2–1 Seiryo-machi, Aoba-ku, Sendai, Miyagi 980–8575, Japan; 2 Department of Anesthesia, Brigham and Women’s Hospital, Harvard Medical School, 75 Francis Street, Boston, MA 02115, United States of America; INRS, CANADA

## Abstract

Podoplanin (PDPN), also known as Aggrus, possesses three tandem repeat of platelet aggregation-stimulating (PLAG) domains in its N-terminus. Among the PLAG domains, sialylated *O*-glycan on Thr52 of PLAG3 is essential for the binding to C-type lectin-like receptor-2 (CLEC-2) and the platelet-aggregating activity of human PDPN (hPDPN). Although various anti-hPDPN monoclonal antibodies (mAbs) have been generated, no specific mAb has been reported to target the epitope containing glycosylated Thr52. We recently established CasMab technology to develop mAbs against glycosylated membrane proteins. Herein, we report the development of a novel anti-glycopeptide mAb (GpMab), LpMab-12. LpMab-12 detected endogenous hPDPN by flow cytometry. Immunohistochemical analyses also showed that hPDPN-expressing lymphatic endothelial and cancer cells were clearly labeled by LpMab-12. The minimal epitope of LpMab-12 was identified as Asp49–Pro53 of hPDPN. Furthermore, LpMab-12 reacted with the synthetic glycopeptide of hPDPN, corresponding to 38–54 amino acids (hpp3854: _38_-EGGVAMPGAEDDVVTPG-_54_), which carries α2–6 sialylated *N*-acetyl-_D_-galactosamine (GalNAc) on Thr52. LpMab-12 did not recognize non-sialylated GalNAc-attached glycopeptide, indicating that sialylated GalNAc on Thr52 is necessary for the binding of LpMab-12 to hPDPN. Thus, LpMab-12 could serve as a new diagnostic tool for determining whether hPDPN possesses the sialylation on Thr52, a site-specific post-translational modification critical for the hPDPN association with CLEC-2.

## Introduction

Podoplanin (PDPN), the endogenous ligand of C-type lectin-like receptor-2 (CLEC-2) [1,2], is highly expressed not only in various tumors including oral cancer, lung cancer, esophageal cancer, malignant brain tumors, mesotheliomas, testicular tumors, and osteosarcoma [3–13], but also in normal cells such as lymphatic endothelial cells and podocytes [14,15]. PDPN is also abundant in lung type I alveolar cells where it is called "T1α" [16]. We previously named PDPN as "Aggrus" because PDPN possesses a platelet aggregation-inducing activity, which is associated with cancer metastasis [3]. Further, PDPN is known as a specific lymphatic endothelial marker [17], and PDPN-CLEC-2 signaling leads to platelet aggregation, which is critical for the embryonic blood-lymphatic vascular separation [18].

Interaction of human PDPN (hPDPN) with CLEC-2 mainly involves Glu47 and Asp48 in the platelet aggregation-stimulating domain-3 (PLAG3) and the **α**2–6 linked sialic acid residue [19]. The sequence motif is conserved among PDPNs of various species [20]. CLEC-2 has a recognition motif in the form ‘‘EDXXXT/S,” where X is any amino acid, and T (or S) contains disialyl-core 1 [21]. In our previous studies, we established that the glycosylation on Thr52 is critical for the binding of hPDPN to CLEC-2, and the sialylated *O*-glycan on Thr52 is required for the platelet aggregating activity of hPDPN [3,21]. Therefore, the detection of site-specific glycosylation on Thr52 is important for determining whether in given pathophysiological conditions, hPDPN is prone to the CLEC-2 binding and has the potential to cause platelet aggregation.

Here, we describe the development and characterization of a new anti-hPDPN mAb, LpMab-12, which specifically binds to glycosylated Thr52, and might serve as a novel modality to study hPDPN-CLEC-2 interaction.

## Materials and Methods

### Cell lines, animals, and tissues

Chinese hamster ovary (CHO)-K1, LN229, HEK-293T, COS-7, and P3U1 were obtained from the American Type Culture Collection (ATCC, Manassas, VA). Human lymphatic endothelial cell (LEC) was purchased from Cambrex (Walkersville, MD). The human glioblastoma cell line, LN319, was donated by Dr. Kazuhiko Mishima (Saitama Medical University, Saitama, Japan). LN229 was transfected with human PDPN plasmids (LN229/hPDPN) using Lipofectamine 2000 (Thermo Fisher Scientific Inc., Waltham, MA) according to the manufacturer’s instructions [22]. LN319/hPDPN-knock out (KO) cells (PDIS-6), HEK-293T/hPDPN-KO cells (PDIS-2), and COS-7/hPDPN-KO cells (PDIS-4) were produced by transfecting CRISPR/Cas plasmids, which targets hPDPN (Sigma-Aldrich Corp., St. Louis, MO), using a Gene Pulser Xcell electroporation system (Bio-Rad Laboratories Inc., Philadelphia, PA). The amplified hPDPN cDNA was subcloned into a pcDNA3 vector (Thermo Fisher Scientific Inc.) and a FLAG epitope tag was added at the C-terminus. Substitution of amino acids to alanine in hPDPN was performed using a QuikChange Lightning site-directed mutagenesis kit (Agilent Technologies Inc., Santa Clara, CA). CHO-K1 cells were transfected with the plasmids using a Gene Pulser Xcell electroporation system (Bio-Rad Laboratories Inc.). P3U1 and CHO-K1 cell lines, and their counterparts transfected with hPDPN were cultured in L-glutamine-containing RPMI 1640 medium (Nacalai Tesque, Inc., Kyoto, Japan), and LN229, LN319, HEK-293T, COS-7 cell lines and their transfected counterparts were cultured in L-glutamine-containing Dulbecco’s Modified Eagle’s Medium (DMEM) medium (Nacalai Tesque, Inc.), supplemented with 10% heat-inactivated fetal bovine serum (FBS; Thermo Fisher Scientific Inc.) at 37°C in a humidified atmosphere of 5% CO_2_ and 95% air. LEC was cultured in endothelial cell medium EGM-2MV supplemented with 5% FBS (Cambrex Corp.). Antibiotics including 100 units/ml of penicillin, 100 μg/ml of streptomycin, and 25 μg/ml of amphotericin B (Nacalai Tesque, Inc.) were added to all media.

Three female BALB/c mice (four-weeks old) were purchased from CLEA Japan (Tokyo, Japan). Animals were housed under pathogen-free conditions. "The Animal Care and Use Committee of Tohoku University" approved the animal experiments described herein. The use of one oral cancer tissue was reviewed and approved by "Tokyo Medical and Dental University Institutional Review Board" [23]. Written informed consent was obtained for the human cancer tissue samples used in this study. The use of human heart tissue sections for immunohistochemical analysis was reviewed and approved by the "Partners Institutional Review Board".

### Hybridoma production

Three BALB/c mice were immunized by intraperitoneal (i.p.) injection of 1 × 10^8^ LN229/hPDPN cells together with Imject Alum (Thermo Fisher Scientific Inc.), as previously described [22]. After several additional immunizations, a booster injection was given i.p. two days before mice were euthanized by cervical dislocation and spleen cells were harvested. The spleen cells were fused with P3U1 cells using PEG1500 (Roche Diagnostics, Indianapolis, IN). The fused cells were grown in RPMI medium with hypoxanthine, aminopterin, and thymidine selection medium supplement (Thermo Fisher Scientific Inc.). The culture supernatants were screened using enzyme-linked immunosorbent assay (ELISA) for binding to recombinant hPDPN purified from LN229/hPDPN cells.

### Enzyme-linked immunosorbent assay (ELISA)

Recombinant hPDPN or glycopeptides were immobilized on Nunc Maxisorp 96-well immunoplates (Thermo Fisher Scientific Inc.) at a concentration of 1 μg/ml for 30 min. After blocking with 1% BSA in 0.05% Tween20/phosphate buffered saline (PBS, Nacalai Tesque, Inc.), the plates were incubated with culture supernatant followed by 1:1000 diluted peroxidase-conjugated anti-mouse IgG or anti-rat IgG (Dako; Agilent Technologies, Inc., Glostrup, Denmark). The enzymatic reaction was conducted with a 1-Step Ultra TMB-ELISA (Thermo Fisher Scientific Inc.). The optical density was measured at 655 nm using an iMark microplate reader (Bio-Rad Laboratories Inc.).

### Western blot analyses

Cell lysates (10 μg) were boiled in sodium dodecyl sulfate (SDS) sample buffer (Nacalai Tesque, Inc.). The proteins were electrophoresed on 5–20% polyacrylamide gels (Wako Pure Chemical Industries Ltd.) and were transferred onto a PVDF membrane (EMD Millipore Corp., Billerica, MA). After blocking with 4% skim milk (Nacalai Tesque, Inc.) in 0.05% Tween20/PBS, the membrane was incubated with 1 μg/ml of LpMab-12, LpMab-7, 1E6 (anti-FLAG; Wako Pure Chemical Industries Ltd.), RcMab-3 (anti-IDH1) [24], or AC-15 (anti-β-actin; Sigma-Aldrich Corp.) and then with peroxidase-conjugated anti-mouse IgG (1:1000 diluted; Dako), and developed with the ImmunoStar LD Chemiluminescence Reagent (Wako Pure Chemical Industries Ltd.) using a Sayaca-Imager (DRC Co. Ltd., Tokyo, Japan).

### Flow cytometry

Cell lines were harvested by brief exposure to 0.25% Trypsin/1 mM EDTA (Nacalai Tesque, Inc.). After washing with PBS, the cells were incubated with LpMab-12 (1 μg/ml) for 30 min at 4°C, followed by the incubation with Oregon Green 488 goat anti-mouse IgG (Thermo Fisher Scientific Inc.). Fluorescence data were collected using a Cell Analyzer EC800 (Sony Corp., Tokyo, Japan).

### Determination of the apparent binding affinity using flow cytometry

LN319 (2 × 10^5^ cells) and LEC (1 × 10^5^ cells) were resuspended in 100 μl of serially diluted LpMab-12 (0.061–100 μg/ml) followed by Oregon Green 488 goat anti-mouse IgG (Thermo Fisher Scientific Inc.). Fluorescence data were collected using a cell analyzer (EC800; Sony Corp.). The apparent dissociation constants (K_D_) were obtained by fitting the binding isotherms using the built-in one-site binding models in GraphPad PRISM 6 (GraphPad software, Inc., La Jolla, CA).

### Immunohistochemical analysis of human heart tissues

Four-μm-thick histologic sections of the myocardium were deparaffinized in xylene, rehydrated, and subjected to 10 min heat-induced antigen retrieval in citric buffer (pH 6.0). Samples were blocked in 10% normal donkey serum (Jackson ImmunoResearch Inc., West Grove, PA) for 30 min at room temperature, incubated with 10 μg/ml of LpMab-12 overnight at 4°C, and then with Alexa Fluor 568-conjugated donkey anti-mouse IgG (Thermo Fisher Scientific Inc.) for 1 h at 37°C. Subsequently, the sections were incubated with goat anti-human LYVE-1 (10 μg/ml; R&D Systems, Inc., Minneapolis, MN) and mouse anti-α-sarcomeric actin (α-SA) (1:200 diluted; Sigma-Aldrich Corp.) for 2 h at 37°C, followed by fluorescein isothiocyanate (FITC)-conjugated donkey anti-goat IgG and Alexa Fluor 647-conjugated donkey anti-mouse IgM (15 μg/ml each; Jackson ImmunoResearch Inc.) and 4',6-diamidino-2-phenylindole dihydrochloride (DAPI) (1 μg/ml; Sigma-Aldrich Corp.) for 1 h at 37°C. The sections were then treated with 1% solution of Sudan Black B (Sigma-Aldrich Corp.) for 30 min at room temperature, and mounted in Vectashield medium (Vector Laboratories, Inc., Road Burlingame, CA). Images were acquired with Olympus FluoView FV100 laser scanning confocal microscope equipped with CCD camera (Bio-Rad Laboratories Inc.).

### Immunohistochemical analyses of oral cancer

Four-μm-thick histologic sections were deparaffinized in xylene and rehydrated. Without antigen retrieval procedure, sections were incubated with 1 μg/ml of LpMab-12 or LpMab-7 for 1 h at room temperature followed by treatment with Envision+ kit (Dako) for 30 min. Color was developed using 3, 3-diaminobenzidine tetrahydrochloride (DAB; Dako), and then the sections were counterstained with hematoxylin (Wako Pure Chemical Industries Ltd.).

### Production of hPDPN glycopeptide

The hPDPN glycopeptide (hpp3854) with a GalNAc residue was purchased from Peptide Institute (Osaka, Japan), and used as an acceptor substrate. For synthesis of the sialylated GalNAc on hpp3854, 25 mM HEPES (pH 7.0) containing 30 μM of acceptor substrate, 10 mM MnCl_2_ (Nacalai Tesque, Inc.), and 250 μM CMP-Neu5Ac (Sigma-Aldrich Corp.) was used. A half volume of purified ST6GalNAcT-I enzyme was added to the reaction mixture and incubated at 37°C for 24 h. The recombinant ST6GalNAcT-I enzyme was bound to anti-FLAG M2 affinity gel (Sigma-Aldrich Corp.). After enzymatic reaction, the resin was removed by filtration using an Ultrafree-MC column (EMD-Millipore). Then, the glycopeptides were purified using a reversed-phase SPE cartridge (ZipTip C18; EMD-Millipore). The other glycopeptides were produced sequentially as previously described [2].

### Assessment of antibody-mediated inhibition of hPDPN binding to hCLEC-2

Inhibition assays were performed by ELISA. The recombinant proteins of hPDPN-Fc [2] and hCLEC-2-Fc [19] were produced in our previous studies. The hPDPN-Fc was immobilized on Nunc Maxisorp 96-well immunoplates (Thermo Fisher Scientific Inc.) at 1 μg/ml for 30 min. After blocking with SuperBlock T20 (PBS) Blocking Buffer, LpMab-2 [22], LpMab-3 [25], LpMab-9 [26], LpMab-12, LpMab-3 + LpMab-12, or isotype control (PMab-32) [27,28] were added at 10 μg/ml for 30 min. The plates were incubated with biotinylated hCLEC-2-Fc (1 μg/ml) followed by 1/1000 diluted peroxidase-conjugated streptavidin (GE Healthcare, Piscataway, NJ). The enzymatic reaction was conducted with a 1-Step Ultra TMB-ELISA (Thermo Fisher Scientific Inc.). The optical density was measured at 655 nm using an iMark microplate reader (Bio-Rad Laboratories Inc.). All data were shown as means ± SD. Statistical analysis by one-way ANOVA was performed using GraphPad Prism 6 (GraphPad Software Inc., La Jolla, CA).

## Results

### Establishment and characterization of a novel anti-hPDPN mAb LpMab-12

**Fig 1:**
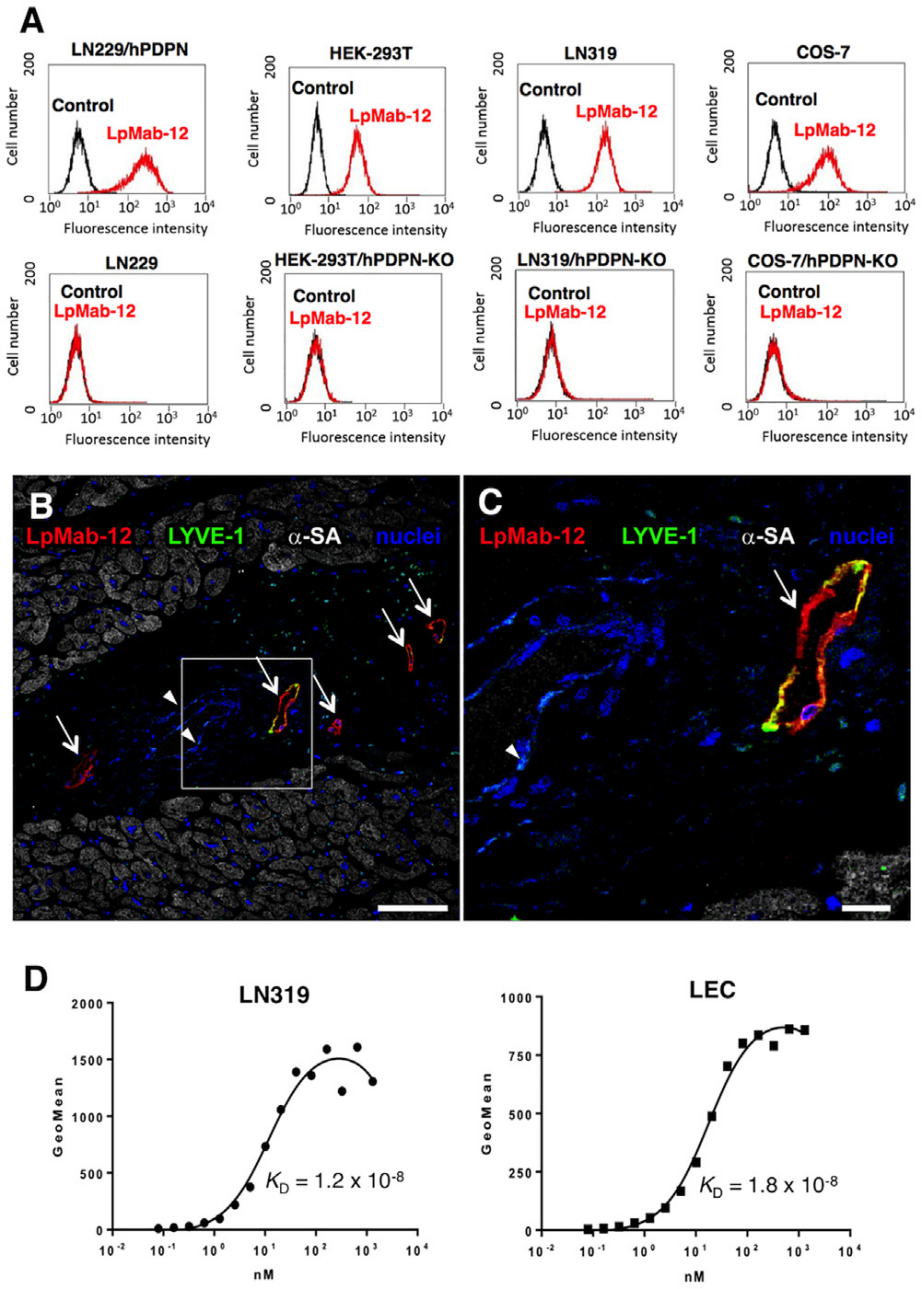
Characterization of an anti-hPDPN mAb LpMab-12. (A) Flow cytometry assessment of LpMab-12 binding to LN229/hPDPN and LN229 cells, endogenous hPDPN-expressing (HEK-293T, LN319, COS-7), and hPDPN-KO cells (HEK-293T/hPDPN-KO, LN319/hPDPN-KO, COS-7/hPDPN-KO). (B, C) Human myocardial samples were indirectly immunolabeled with LpMab-12 (red), the lymphatic endothelial epitope, LYVE-1 (green), and the myocytes were stained with anti-α-sarcomeric actin (α-SA) (grey). Nuclei were counterstained with DAPI (blue). Arrows, lymphatic endothelial cells; arrowheads, vascular endothelial cells of an artery. Scale bars: 100 μm (B) and 20 μm (C). Note the preferential labeling of lymphatic endothelium by LpMab-12. (D) Determination of apparent binding affinity against LN319 and LEC by flow cytometry. The apparent dissociation constants (*K*_D_) were obtained by fitting the binding isotherms using the built-in one-site binding models.

We immunized mice with hPDPN-expressing LN229 glioma cells (LN229/hPDPN), which possess cancer-type glycan patterns including highly sulfated polylactosamine and aberrant sialylation [22]. Spleen cells were harvested and fused with P3U1 cells. Selection of hybridoma was performed using ELISA and flow cytometry, and a novel anti-hPDPN LpMab-12 mAb (mouse IgG_1_, kappa) was developed. Fig 1A shows that LpMab-12 reacted with LN229/hPDPN and endogenous PDPN (HEK-293T, LN319, COS-7), and did not react with LN229 and PDPN-KO cells (HEK-293T/hPDPN-KO, LN319/hPDPN-KO, COS-7/hPDPN-KO), indicating that LpMab-12 reliably detects hPDPN by immunohistochemistry.

As shown in Fig 1B and 1C, lymphatic endothelial cells, identified by a lymphatic marker lymphatic vessel endothelial hyaluronan receptor-1 (LYVE-1), were clearly stained by LpMab-12 in myocardial samples from human hearts, indicating that LpMab-12 is applicable for reliably detecting hPDPN by immunohistochemistry.

Using flow cytometry analysis, apparent dissociation constant of LpMab-12 was determined to be 1.2 × 10^−8^ M for LN319 and 1.8 × 10^−8^ M for LEC, suggesting that the binding affinity of LpMab-12 is comparable with previously established anti-hPDPN mAbs [22] for hPDPN-expressing cancer cells and normal cells (Fig 1D).

**Fig 2:**
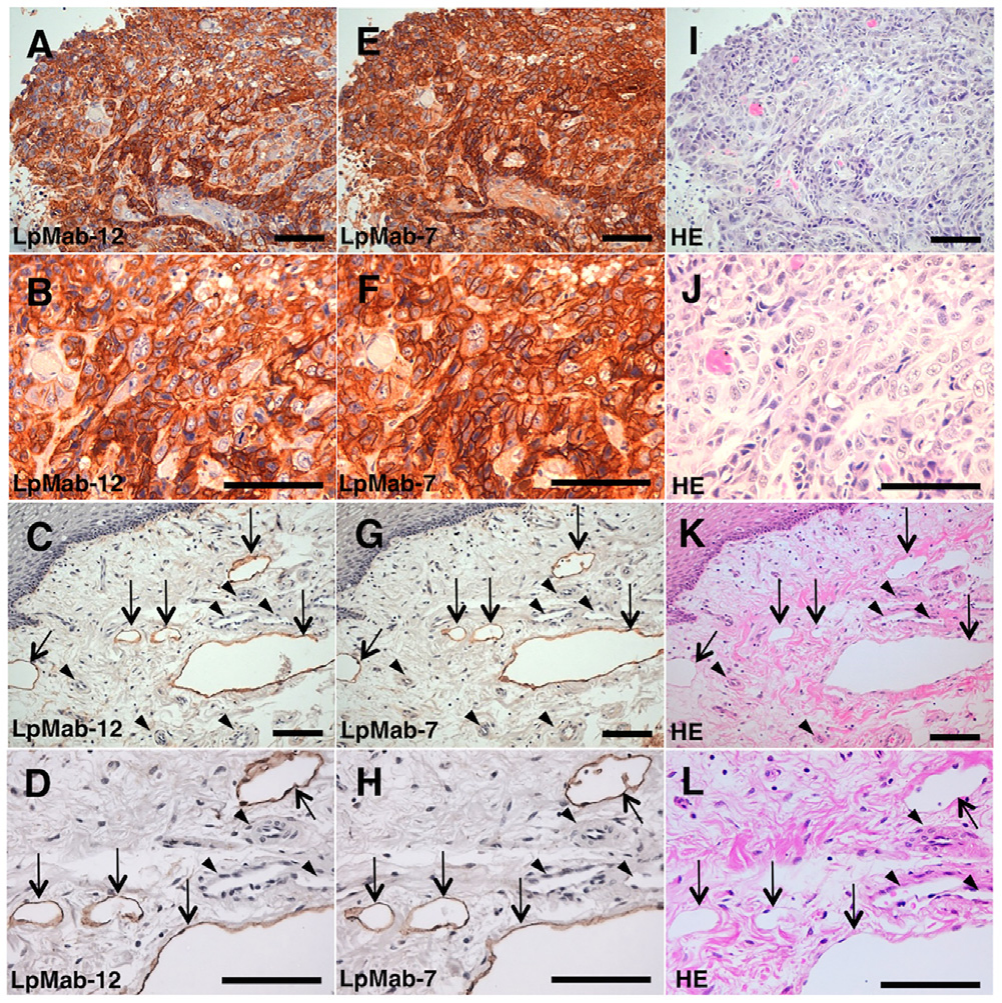
Immunohistochemical analysis of the oral cancer and heart tissue samples using LpMab-12 and LpMab-7. Serial sections of the tissues with oral cancer were incubated with LpMab-12 (A-D) or LpMab-7 (E-H), followed by the development with the EnVision+ kit and counterstaining with hematoxylin, or the HE staining (I-L). Arrows, lymphatic endothelial cells; arrowheads, vascular endothelial cells. Scale bars: 100 μm. LpMab-12 stains lymphatic vessels with high efficiency, similarly to LpMab-7.

To confirm the utility of LpMab-12 for immunolabeling of tissues, we compared the reactivity of LpMab-12 with LpMab-7, the most sensitive anti-hPDPN mAb for this type of analysis [29]. Both LpMab-12 (Fig 2A and 2B) and LpMab-7 (Fig 2E and 2F) strongly stained tumor cells in a membranous/cytoplasmic-staining pattern. Lymphatic vessels were immunolabeled clearly without background by LpMab-12 (Fig 2C and 2D) and LpMab-7 (Fig 2G and 2H). Blood vessels were not stained by both mAbs (Fig 2C, 2D, 2G and 2H).

### Epitope mapping

**Fig 3:**
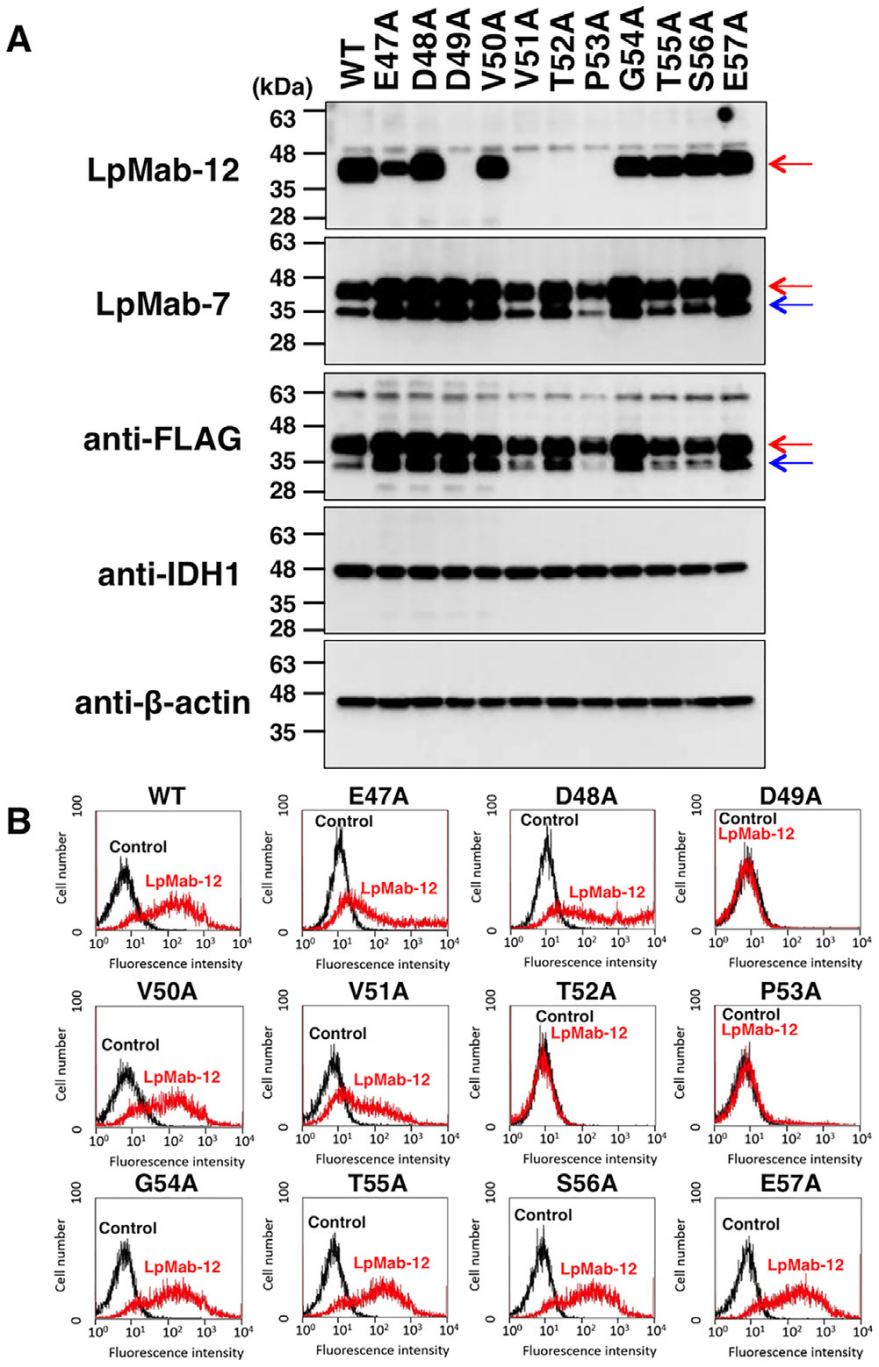
Epitope mapping of LpMab-12 by Western blot analysis and flow cytometry. (A) CHO-K1 cells were transfected with a plasmid expressing wild-type hPDPN with the FLAG-tag added to the C-terminus (WT), or the FLAG-tag hPDPN containing a point mutation in the sequence E47A-E57A, as indicated in the figure. Total cell lysates from the transfected cell lines were analyzed by Western blot with LpMab-12 or LpMab-7, as a positive control for hPDPN expression. Immunoblot with anti-FLAG antibody was also used as well to establish the expression of exogenous hPDPN. Anti-IDH1 and anti-β-actin mAbs were used as internal controls to show that total proteins are equal protein load. Red arrow, 40-kDa; blue arrow, 30-kDa. (B) CHO-K1 cells transfected as in (A) were analyzed by flow cytometry using indirect immunolabeling with LpMab-12. Cells exposed to the secondary anti-mouse IgG only were used as a negative control (Control).

To determine the critical epitope for the LpMab-12 interaction with hPDPN, we compared the mAb binding to the hPDPN carrying different point mutations. Using Western blot, we found that LpMab-12 did not detect protein sequences with the following amino acid substitutions: D49A, V51A, T52A, and P53A (Fig 3A).

In agreement, flow cytometry analysis demonstrated that LpMab-12 did not react with D49A, T52A, and P53A mutant proteins (Fig 3B) Thus, our results indicate that the epitope of LpMab-12 is Asp49-Pro53. In our previous study we established that the sialylated *O*-glycan on Thr52 is critical for platelet aggregating activity of hPDPN [21]. Therefore, the data point that the epitope of LpMab-12 contains the sialylated *O*-glycan on Thr52 in the Asp49-Pro53 sequence of hPDPN.

**Fig 4:**
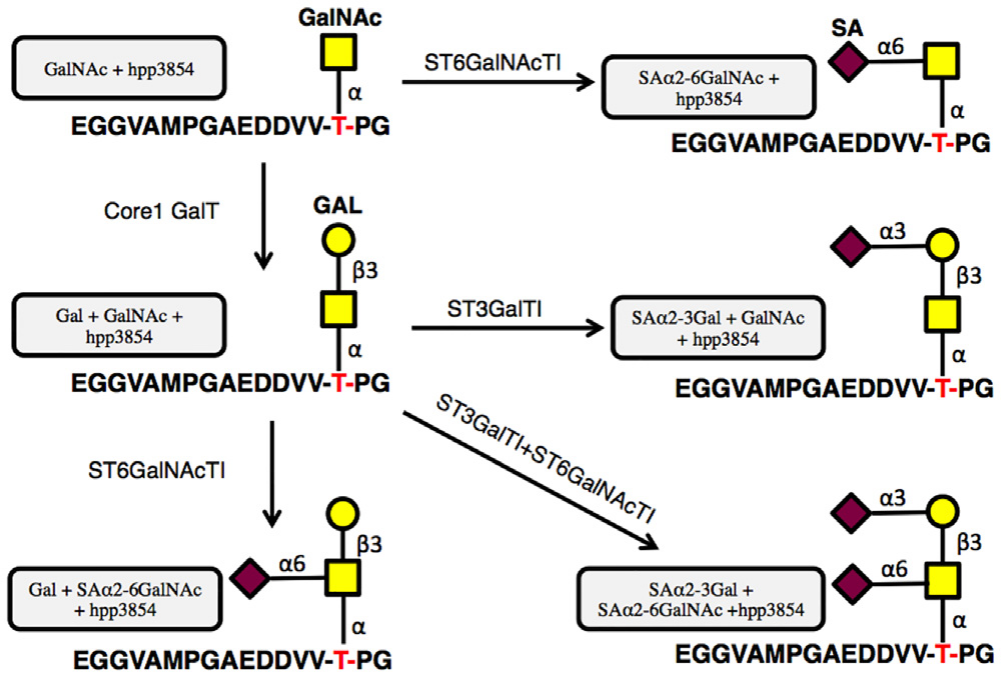
Binding assay of LpMab-12 against sialylated glycopeptide of hPDPN using ELISA. Strategy for the sialylated glycopeptide synthesis. SA, sialic acid; Gal, galacose; GalNAc, *N*-acetyl-_D_-galactosamine.

**Fig 5:**
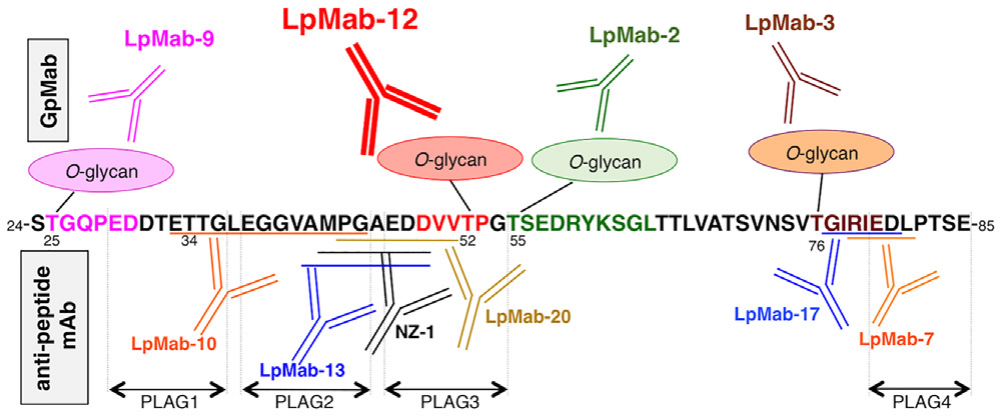
Schematic summary of the epitopes for several anti-hPDPN mAbs. Glycosylation sites are shown (*O*-glycan). Numbers indicate amino acid position. GpMab, anti-glycopeptide mAb; PLAG, platelet aggregation-stimulating.

To further clarify the essential epitope of LpMab-12, especially the essential glycan structure detected by LpMab-12, we synthesized several glycopeptides of hPDPN, which include the PLAG2 and PLAG3 domains (Fig 4). Specifically, we generated SAα2-6GalNAc + hpp3854; Gal + GalNAc + hpp3854; SAα2-3Gal + GalNAc + hpp3854; Gal + SAα2-6GalNAc + hpp3854; and SAα2-3Gal + SAα2-6GalNAc + hpp3854. LpMab-12 detected SAα2-6GalNAc + hpp3854, Gal + SAα2-6GalNAc + hpp3854, and SAα2-3Gal + SAα2-6GalNAc + hpp3854 (Table 1). LpMab-9, the epitope of which was identified as residues 25–30 of hPDPN [26], did not react with any glycopeptides of hpp3854. In contrast, LpMab-13 and LpMab-20, which were recently established using CasMab technology [30], recognized all the glycopepties of hpp3854. Collectively, these data indicate that the essential epitope of LpMab-12 is _49_-DVVT(SAα2-6GalNAc)P-_53_ (Fig 5).

**Table 1:** The reaction of LpMab-12 against glycopeptides of hPDPN.

	Anti-hPDPN mAbs			
Glycopeptides	LpMab-9	LpMab-12	LpMab-13	LpMab-20
GalNAc + hpp3854	-	-	+++	+++
Gal + GalNAc + hpp3854	-	-	+++	+++
SAα2-6GalNAc + hpp3854	-	+++	+++	+++
SAα2-3Gal + GalNAc + hpp3854	-	-	+++	+++
Gal + SAα2-6GalNAc + hpp3854	-	+++	+++	+++
SAα2-3Gal + SAα2-6GalNAc + hpp3854	-	+++	+++	+++

+++, 0.5≦OD655; -, negative

### Neutralization assays of hPDPN and CLEC-2

**Fig 6:**
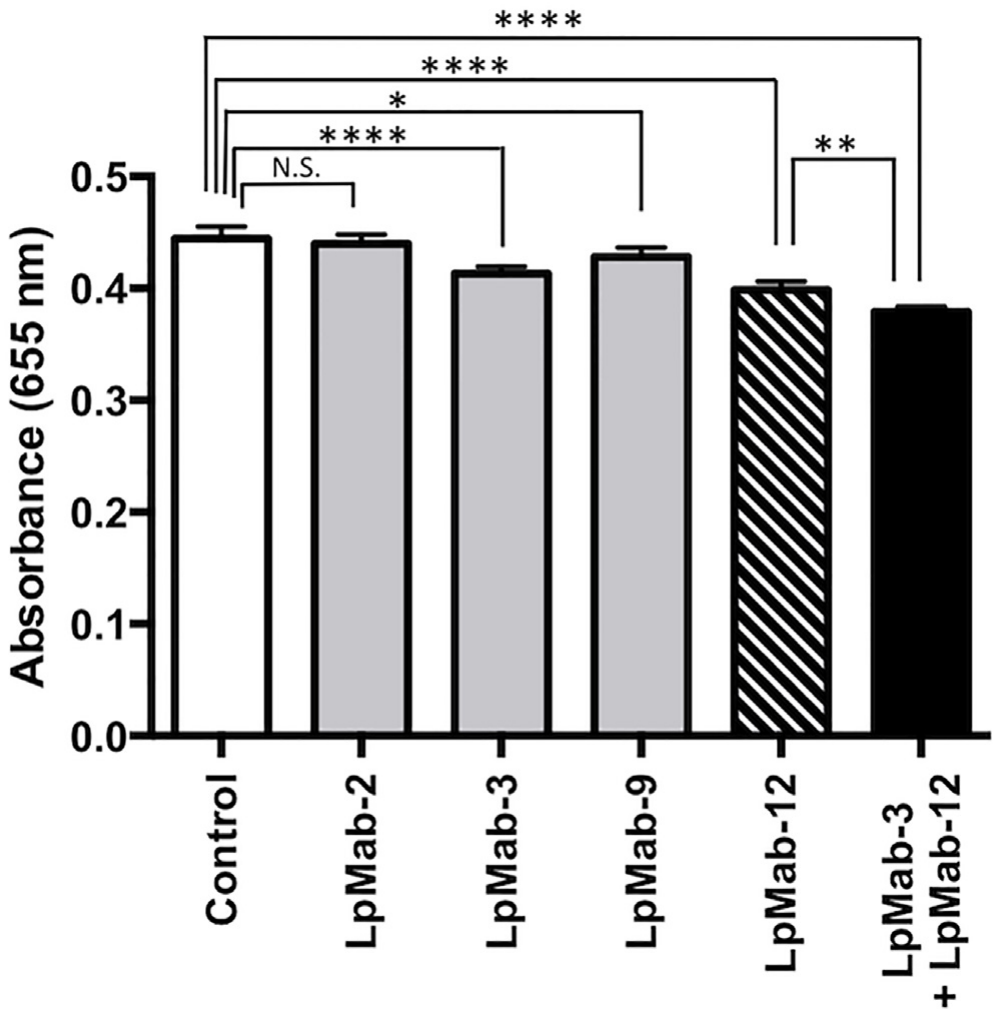
The hPDPN-hCLEC-2 interaction was reduced by LpMab-12. Inhibition assay was performed using ELISA. Recombinant immobilized hPDPN-Fc was incubated with PMab-32 (Control), LpMab-2, LpMab-3, LpMab-9, LpMab-12, or LpMab-3 + LpMab-12, followed by sequential exposure to biotinylated hCLEC-2-Fc and peroxidase-conjugated streptavidin. The enzymatic reaction was conducted with a 1-Step Ultra TMB-ELISA. The optical density was measured at 655 nm. Each data bar represents the average of five independent wells. Error bars show standard deviation (SD). N.S., not significant, * *P* < 0.05, ** *P* < 0.01, ****P < 0.0001, as determined by one-way ANOVA.

Finally, we determined whether LpMab-12 inhibits hPDPN-CLEC-2 interaction using ELISA. Additional anti-hPDPN mAbs such as LpMab-2, LpMab-3, and LpMab-9 were employed as controls. Similarly to LpMab-12, LpMab-2 [22], LpMab-3 [25] and LpMab-9 [26] include both peptide and glycan as their epitopes; accordingly, we collectively named these mAbs as anti-glycopeptide mAbs (GpMabs). Anti-rabbit PDPN antibody PMab-32 was used as an additional negative control, since it is of the same isotype (mouse IgG_1_, kappa) with LpMab-12 [27]. As shown in Fig 6, LpMab-12 impaired the binding of hCLEC-2-Fc to hPDPN-Fc (10.3% inhibition), whereas LpMab-2 did not affect the hPDPN/hCLEC-2 interaction. Interestingly, LpMab-3, the epitope of which includes Thr76 of hPDPN [25] also moderately reduced the hPDPN/hCLEC-2 binding (7.1% inhibition); and LpMab-9, the epitope of which includes Thr25 of hPDPN, impaired the hPDPN/hCLEC-2 interaction to a lesser extent (3.6% inhibition). Importantly, the combination of LpMab-12 and LpMab-3 reduced the hPDPN/hCLEC-2 interaction more effectively (14.7% inhibition) than either LpMab-12 or LpMab-3 alone, indicating that a combination of several sialic acids in the hPDPN protein might be important for its optimal interaction with hCLEC-2 in this *in vitro* assay.

## Discussion

As of today, almost all anti-hPDPN mAbs produced using conventional methods react with the non-glycosylated peptide spanning the PLAG1-3 domains [7,12,31] or PLAG4 domain [32]. Our group also produced numerous mAbs against mouse, rat and rabbit PDPN proteins [27,33,34]. Rabbit polyclonal antibodies, which were reported by Matsui *et al*., also recognize PLAG1-3 domains, which are shown to be immunodominant antigenic sites of PDPN [35]. Recently, we established the CasMab technology for the production of cancer-specific mAbs and anti-glycopeptide mAbs (GpMabs). Using CasMab platform, we generated multiple mAbs, including LpMab-2, LpMab-3, LpMab-7, LpMab-9, LpMab-10, and LpMab-17, which target different epitopes of hPDPN [22,23,25,26,29,36–38]. Furthermore, using CasMab approach, we produced mAbs that detect residue-specific *O*-glycosylation in hPDPN: LpMab-2 on Thr55/Ser56, LpMab-3 on Thr76, and LpMab-9 on Thr25. Although the glycosylation on Thr52 is the most critical for the binding of hPDPN to CLEC-2 and platelet aggregating-activity of hPDPN [2,19], no GpMab against Thr52-containing epitope has been developed. The direct detection of glycosylation on Thr52 using specific mAb might be implemented for investigating the function of hPDPN or clinical diagnosis.

In this study, we successfully developed LpMab-12 (mouse IgG_1_, kappa), which specifically detects the glycosylation on Thr52 of hPDPN by flow cytometry (Figs 1 and 3), Western blot (Fig 3), and immunohistochemical analysis (Figs 1 and 2). Because this modification was previously shown to be of critical importance for hPDPN-CLEC-2 interaction [2,19], we hypothesized that LpMab-12 might interfere with the hPDPN-binding to CLEC-2. We found that LpMab-12 only partially and weakly reduced the hPDPN binding to hCLEC-2, yet with a higher efficiency than the other anti-hPDPN glycopeptide mAbs (GpMabs), such as LpMab-3 and LpMab-9 (Fig 6). These results indicate that hCLEC-2 might interact with several sialic acids attached to Ser/Thr of hPDPN. Indeed, a novel platelet aggregation-stimulating domain-4 (PLAG4) of hPDPN (Fig 5) was recently suggested [32], further supporting the notion that complex interactions might be required for an optimal association of hPDPN with hCLEC-2.

Our data show that LpMab-12 is advantageous for the use for hPDPN detection in fixed paraffin-embedded tissue sections, since, unlike other anti-hPDPN antibodies, including LpMab-2 and LpMab-3 [22,25], or D2-40 and 18H5 [31], LpMab-12 does not necessitate antigen retrieval (Fig 2). Further, in most PDPN immunolabeling protocols, the antibodies have to be used at a concentration of 1 μg/ml or higher [22,25,31], whereas relatively low concentrations of LpMab-12 (less than 0.1 μg/ml) are sufficient to detected the lymphatic endothelial cells in fixed samples (data not shown).

Lec2 mutant of CHO cells lacks a CMP-sialic acid transporter, and is not able to add sialic acid to glycans. In contrast, Lec8 mutant of CHO cells lacks a UDP-Gal transporter and is not able to add Gal to glycans [39]. Our results show that LpMab-12 detects hPDPN with sialylated *O*-GalNAc (Fig 4 and Table 1); therefore, LpMab-12 did not react with Lec2/hPDPN (S1B Fig). Surprisingly, we observed that LpMab-12 did not react with Lec8/hPDPN cells even at relatively high concentrations of 10 μg/ml or 100 μg/ml (S1C Fig). Future studies are warranted to determine the reason for the deficiency in *O*-GalNAc sialylation in Lec8/hPDPN.

## Conclusion

Our study suggests that LpMab-12 is useful for determining whether hPDPN possesses the site-specific sialylation on Thr52, an important post-translational modification for the association of hPDPN with CLEC-2 and activation of platelet aggregation. Furthermore, the combination of different epitope-specific mAbs, especially GpMabs, might be advantageous for the PDPN-targeting therapies or disease diagnosis.

## Supporting Information

S1 Fig (TIFF)

S1 File (DOCX)

## Abbreviations

mAb: monoclonal antibody
PLAG: platelet aggregation-stimulating
GalNAc: *N*-acetyl-_D_-galactosamine
GpMab: anti-glycopeptide mAb
CLEC-2: C-type lectin-like receptor-2
LEC: lymphatic endothelial cell
