# Supplementary figures and images for: LpMab-12 Established by CasMab Technology Specifically Detects Sialylated O-Glycan on Thr52 of Platelet Aggregation-Stimulating Domain of Human Podoplanin
Source: PLoS One. 2016 Mar 31;11(3):e0152912. doi: 10.1371/journal.pone.0152912 (PMC4816300; doi:10.1371/journal.pone.0152912)

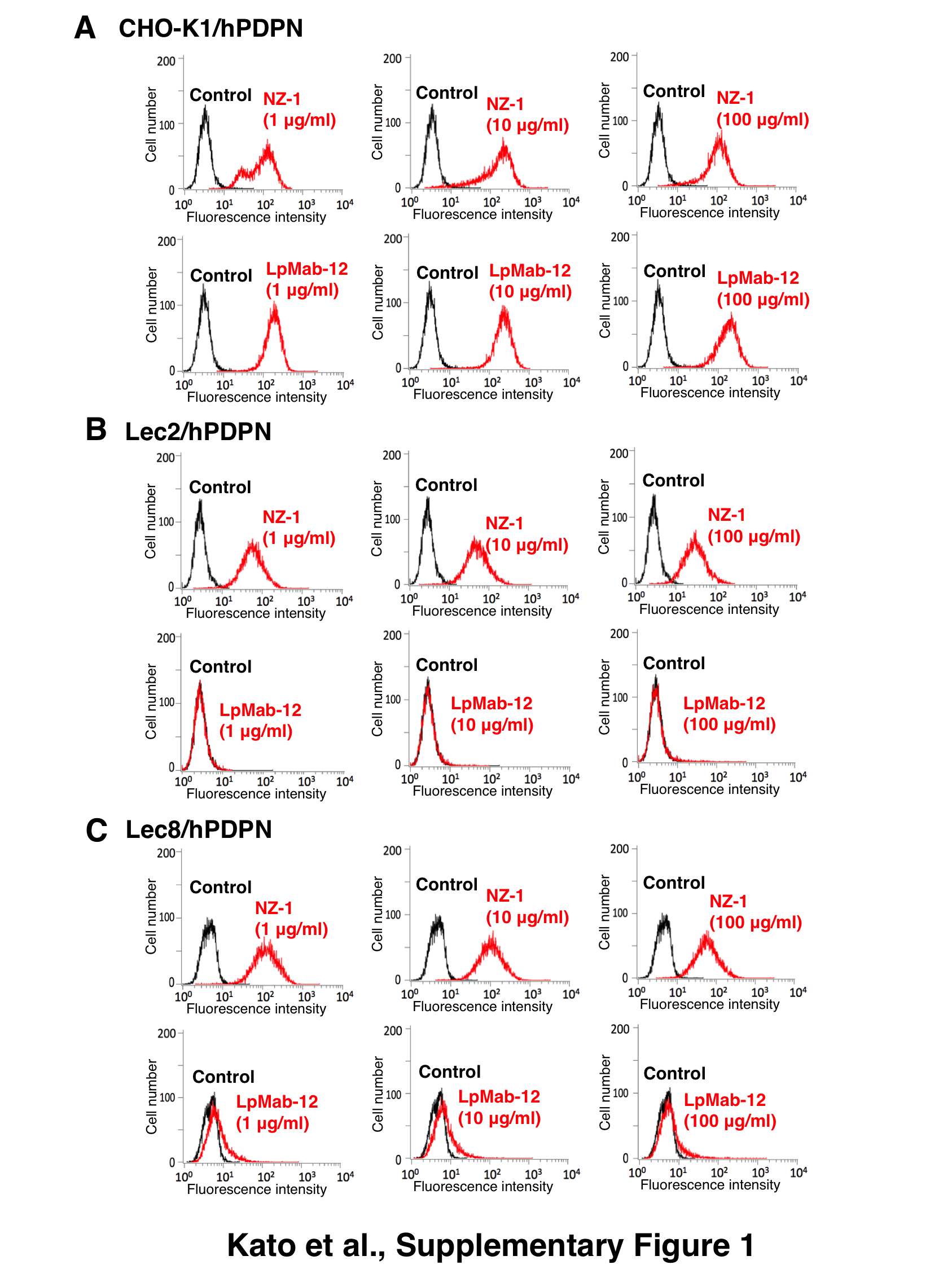

Supplement: S1 Fig — (TIFF) [file pone.0152912.s001.tiff]
