# Supplementary material for: LpMab-12 Established by CasMab Technology Specifically Detects Sialylated O-Glycan on Thr52 of Platelet Aggregation-Stimulating Domain of Human Podoplanin
Source: PLoS One. 2016 Mar 31;11(3):e0152912. doi: 10.1371/journal.pone.0152912 (PMC4816300; doi:10.1371/journal.pone.0152912)
Supplement: S1 File — (DOCX) [file pone.0152912.s002.docx]

**Supplementary information**

**Materials and methods**

Cell lines, animals, and tissues

Chinese hamster ovary (CHO)-K1, glycan-deficient CHO cell lines (Lec2 and Lec8) were obtained from the American Type Culture Collection (ATCC, Manassas, VA). CHO-K1, Lec2, and Lec8 were transfected with human PDPN plasmids (CHO/hPDPN, Lec2/hPDPN, Lec8/hPDPN) using Lipofectamine 2000 (Thermo Fisher Scientific Inc., Waltham, MA). CHO-K1, Lec2, Lec8 cell lines, and their counterparts transfected with hPDPN were cultured in RPMI 1640 medium (Nacalai Tesque, Inc., Kyoto, Japan), supplemented with 10% heat-inactivated fetal bovine serum (FBS; Thermo Fisher Scientific Inc.) at 37°C in a humidified atmosphere of 5% CO_2_ and 95% air. L-proline (0.04 mg/ml) was added for Lec2 and Lec8. Antibiotics including 100 units/ml of penicillin, 100 μg/ml of streptomycin, and 25 μg/ml of amphotericin B (Nacalai Tesque, Inc.) were added to all media.

Flow cytometry

Cell lines were harvested by brief exposure to 0.25% Trypsin/1 mM EDTA (Nacalai Tesque, Inc.). After washing with PBS, the cells were incubated with LpMab-12 (1, 10, or 100 μg/ml) or NZ-1 (1, 10, or 100 μg/ml) for 30 min at 4°C, followed by the incubation with Oregon Green 488 goat anti-mouse IgG or anti-rat IgG (Thermo Fisher Scientific Inc.). Fluorescence data were collected using a Cell Analyzer EC800 (Sony Corp., Tokyo, Japan).

**Figure legends**

**S1 Fig** Flow cytometry analysis of LpMab-12 binding to hPDPN. The hPDPN-transfected cells (CHO-K1/hPDPN (A), Lec2/hPDPN (B), Lec8/hPDPN (C)) were incubated with LpMab-12 (1, 10, or 100 μg/ml), NZ-1 (1, 10, or 100 μg/ml) or PBS (Control) for 30 min at 4°C, followed by Oregon Green 488 conjugated anti-mouse or rat IgG.
